# Predicting chronic subdural hematoma risk in elderly patients with mild traumatic brain injury

**DOI:** 10.1007/s00701-026-06788-5

**Published:** 2026-02-18

**Authors:** Mai Ofri, Amit Azriel, Lotem Kahati, Nave Paran, Simon Esbit, Yuval Sufaro, Noam Barda

**Affiliations:** 1 Faculty of Health Sciences, Ben-Gurion University of the Negev, Beer-Sheva, Israel; 2 Joyce and Irving Goldman Medical School, Faculty of Health Sciences, Ben-Gurion University of the Negev, Beer-Sheva, Israel; 3 Clinical Research Center, Soroka University Medical Center, Beer-Sheva, Israel; 4 Department of Neurosurgery, Soroka University Medical Center, Beer Sheva, Israel; 5 Medical School for International Health, Ben-Gurion University of the Negev, Beer-Sheva, Israel; 6 Department of Epidemiology, and Community Health Sciences, Ben-Gurion University of the Negev, BiostatisticsBeer-Sheva, Israel; 7 Department of Software and Information Systems Engineering, Ben-Gurion University of the Negev, Beer-Sheva, Israel

**Keywords:** Traumatic Brain Injury, Chronic Subdural Hematoma, Prediction model, Risk Factors

## Abstract

**Purpose:**

Mild traumatic brain injury (TBI) is a frequent presentation in the emergency department. Chronic subdural hematoma (CSDH), most often caused by TBI, is increasing in incidence due to population aging and widespread anticoagulant use. Identifying mild TBI patients at high-risk of CSDH remains challenging. This study aimed to identify risk factors and to create a simple prediction model for CSDH following mild head injury.

**Methods:**

We conducted a historical cohort study including patients aged 65 and older who presented with mild TBI to the emergency department of a large tertiary district medical center between 2000 and 2021. The primary outcome was the development of CSDH within 2–12 weeks after the head injury. A prediction model including demographics, comorbidities, and chronic-medication-use was developed.

**Results:**

Of 7,246 eligible patients, 92 developed CSDH. Age (OR = 1.03, 95% CI: 1.00–1.06, per one-year increase), male sex (OR = 2.31, 1.48–3.59), renal failure (OR = 2.16, 1.27–3.67), chronic use of anticoagulant medications (OR = 1.64, 0.77–3.49), and pathological computed tomography (CT) at presentation (OR = 12.73, 8.19–19.79), were most strongly associated with development of CSDH. Among patients who developed CSDH, 58% had pathological CT findings at presentation. A simple score-based risk model had an area under the receiver operating characteristic curve of 0.76 (0.67–0.85).

**Conclusions:**

Pathological CT findings following mild TBI were the strongest predictor for CSDH. Male sex, older age, and renal failure were also strong predictors. The prediction model allows efficient bedside risk stratification and opens the possibility for targeted surveillance protocols in high-risk populations.

**Supplementary Information:**

The online version contains supplementary material available at 10.1007/s00701-026-06788-5.

## Introduction

Traumatic brain injury (TBI) is a challenging public health problem [19]. According to the Centers for Disease Control and Prevention, 2.5 million TBI-related emergency department (ED) visits were documented in the United States in 2014. Adults aged 75 years and older have the highest rate of TBI-associated ED visits (1,682‏ per 100,000 population) [15].

It is estimated that 70–90% of all TBIs are mild, although the precise proportion is hard to ascertain because some individuals with mild TBI do not seek medical attention [8, 16]. Computed tomography (CT) images are often unremarkable in this group, although some individuals may have CT abnormalities [8]. Patients with mild TBI and an abnormal CT scan are sometimes referred to as having “complicated mild TBI”, which may be associated with unfavorable outcomes [8, 10].

With the aging of the population and the increased use of antithrombotic agents, the incidence of chronic subdural hematoma (CSDH) is rising worldwide [5, 14, 22, 24]. The majority (50–77%) of CSDH patients have a history of documented head trauma in the weeks preceding the onset of signs and symptoms of CSDH [6, 14]. Though CSDH is generally considered a non-life-threatening event, recent studies reported poor long-term functional, mental, and cognitive outcomes following surgical drainage [13]. The incidence of CSDH ranges from 1.72 to 20.6 per 100,000 persons per year, with a higher incidence observed in the elderly [22].

Little is known about specific risk factors for developing CSDH following minor head trauma, although CSDH has at times been associated with trauma, older age, male sex, and antithrombotic agents (antiplatelets and anticoagulants) [5, 11, 22]. The higher incidence of CSDH seen worldwide is thought to be influenced by the increasing age of the population and the more common use of antithrombotic agents [5, 14]. This trend is projected to continue.

To date, no validated clinical diagnostic rules or prediction models exist to stratify the patient population into low- and high-risk groups. The objectives of this study were to evaluate risk factors for the development of CSDH following mild TBI in the elderly and to derive a validated prediction model suitable for bedside use.

## Methods

### Data and setting

This study is based on the electronic health records of the Soroka University Medical Center (SUMC), integrated with the community electronic medical records of “Clalit” Health Services (CHS). CHS is the largest integrated payer-provider medical organization in Israel, insuring ~ 5,000,000 individuals and providing a full variety of medical services. SUMC is a 1,191-bed tertiary medical center, part of the CHS hospital network, and is the only tertiary hospital in southern Israel, providing comprehensive acute care and follow-up treatment.

The electronic health record of SUMC contains all aspects of patient care, including demographic data, emergency department visits and hospital admissions, patients’ diagnoses, medications, and imaging findings. The integration with the electronic health records of CHS adds data regarding chronic medications and diagnoses.

### Study design and variables

**Fig. 1:**
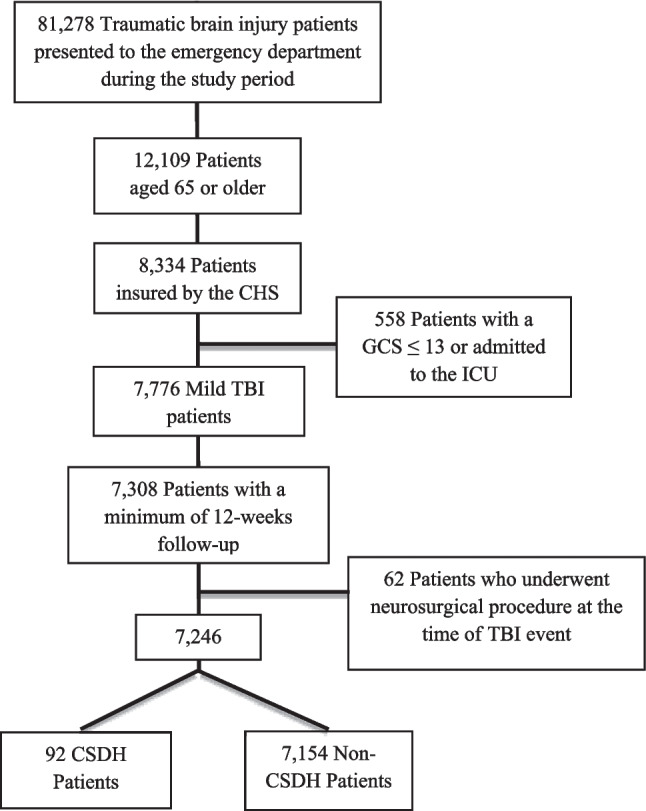
Study population flowchart showing inclusion and exclusion criteria. TBI: Traumatic brain injury; CHS: “Clalit” health services; GCS: Glasgow coma scale; ICU: Intensive care unit; CSDH: Chronic subdural hematoma

This is a historical cohort study. Individuals were included in the study if they presented to the ED at SUMC between January 1, 2000, and October 14, 2021, with a diagnosis of head trauma, and a documented Glasgow Coma Scale (GCS) of 14–15. Patients with missing GCS documentation who were discharged from the ED were presumed to have sustained a mild injury and were also included in this study. In addition to these criteria, patients had to have been insured by CHS, be aged 65 and older, and have either completed the follow-up period or experienced an outcome within the follow-up period, as shown in Fig. 1. To ensure we had access to comprehensive chronic medication data (particularly antithrombotic treatment), we included only patients insured by CHS. We excluded specific groups to maintain the integrity of our findings; ICU patients were removed as their primary treatment focus may not have been their TBI. Patients who underwent an urgent neurosurgical intervention for a subdural hematoma were excluded since subsequent CSDH development could be a surgical outcome, not an outcome of the head injury itself.

Patients were identified as having TBI based on diagnosis codes for traumatic brain injury (Supplementary Table S1). Chronic subdural hematoma (CSDH) was defined as occurring 2–12 weeks after the ED visit, confirmed by CT, and documented using International Classification of Diseases (ICD-9) codes. Patients who underwent a subdural hematoma evacuation (ICD-9 codes listed in Supplementary Table S1) within 2–12 weeks following the initial head injury (i.e., surgically treated CSDH) were included in the overall CSDH group. Accordingly, patients were classified as having CSDH based on either an ICD-9 diagnostic code for CSDH or a surgical evacuation within the specified time window. Patients were followed until either an outcome occurred or for 12 weeks following the injury.

Predictors were chosen based on domain expertise and to ensure adequate statistical power to detect their expected effect. Predictors included demographics (age and sex), comorbidities (diabetes, ischemic heart disease, hypertension, renal failure, dyslipidemia, and atrial fibrillation), pre-existing antiplatelet or anticoagulant medications, and pathological head CT findings from the index event. Pathological head CT at the index TBI event was defined based on diagnosis codes recorded during the event that indicated intracranial hematomas or any type of skull fracture (Supplementary Table S1).

### Statistical analysis

Study variables were summarized using the median and interquartile range (IQR) for continuous variables or counts and proportions for categorical variables. For each variable, we compared patients with and without CSDH using appropriate statistical tests (Wilcoxon’s Rank-Sum test for continuous variables and the Chi-square test for categorical variables). An alpha value of 5% was chosen as the threshold for statistical significance in two-sided testing.

**Fig. 2:**
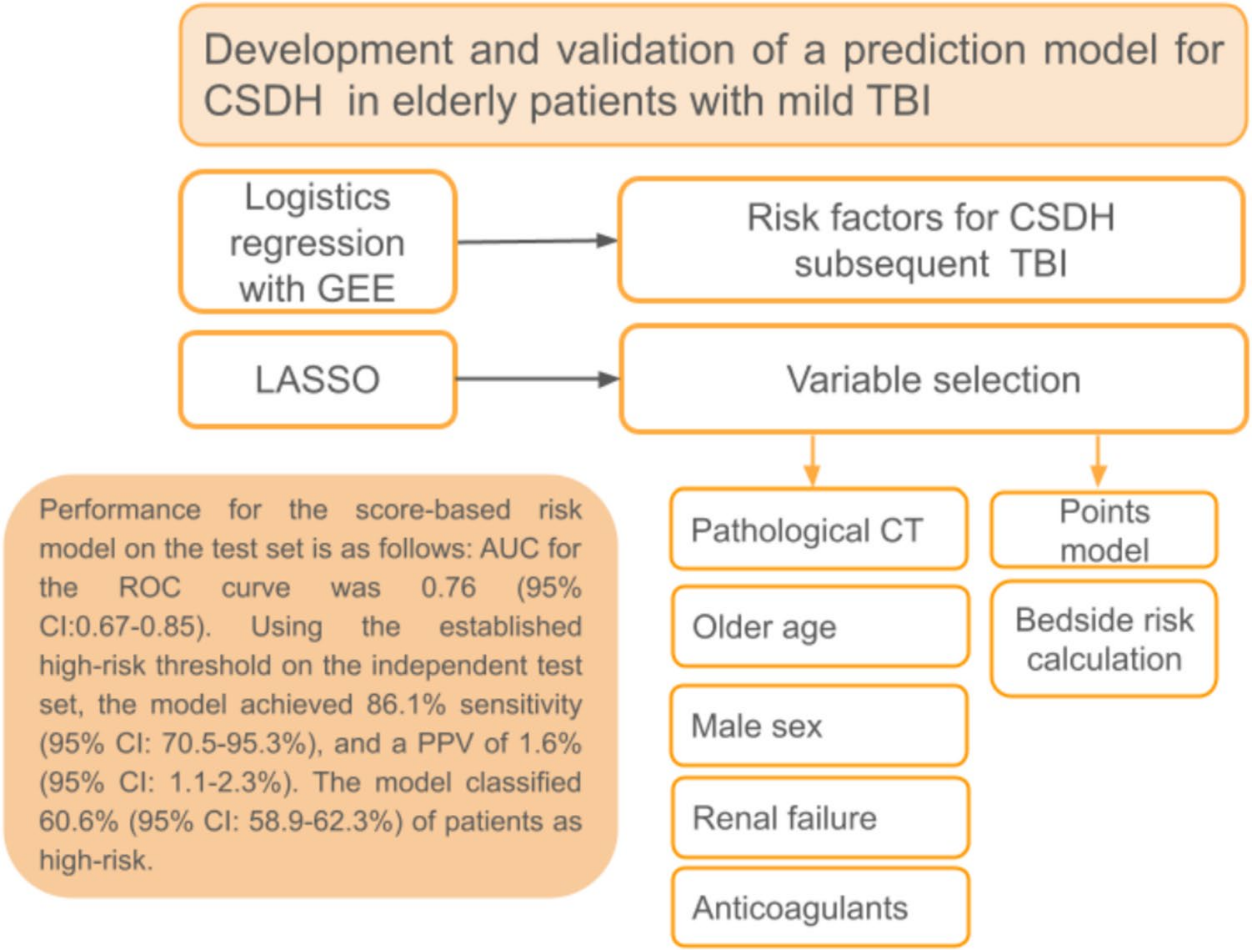
Methodological flowchart showing model development using logistic regression with GEE and LASSO for variable selection. CSDH: Chronic subdural hematoma; TBI: Traumatic brain injury; GEE: Generalized estimating equations; LASSO: Least absolute shrinkage selection operator; AUC: Area under the curve; ROC: Receiver operating characteristics curve; PPV: Positive predictive value; CI: Confidence interval; CT: Computed tomography

As presented in Fig. 2, we identified risk factors by fitting a multivariable logistic regression model. Prior to model fitting, multicollinearity was assessed by calculating the variance inflation factor (VIF). Since all VIF values were less than 2.5, no highly associated variables were removed. The unit of observation was a single mild TBI event; thus, some patients appeared more than once in the analysis set. To account for this, we analyzed the data as hierarchical using a generalized estimating equation (GEE) with an exchangeable working correlation matrix. Effects are expressed as exponentiated model coefficients, interpreted as odds ratios (OR).

Feature selection for the prediction model was performed using LASSO, with the regularization parameter (δ) selected using K-fold cross-validation (K = 10). We included in the LASSO model one case per patient – if a patient had experienced a CSDH event, we included the corresponding injury; otherwise, we included a random injury.

Following feature selection, we divided the data into train and test sets, at a ratio of 0.65:0.35, respectively. We then fit a logistic regression model to the training set using GEE, with the selected features. The model coefficients were rounded and multiplied by five to create a simplified scoring rule for clinical use.

Scoring was initially performed on the train set. Model performance was evaluated across multiple discrimination thresholds for sensitivity, percent positives, and the positive predictive value (PPV). The threshold for the high-risk group was chosen via consultation with domain experts, who suggested that a sensitivity of over 80% was required for a clinically meaningful prediction model. Finally, the scoring rule was applied to the independent test set, and model performance was estimated, including a receiver operating characteristic curve (ROC), sensitivity, PPV, and the percentage of patients predicted at high risk. Confidence intervals (CI) for sensitivity, specificity, positive, and negative predictive value were estimated using the exact binomial test [1]. Confidence intervals for the area under the curve (AUC) were estimated using the DeLong method [3]. For each predicted risk level (by number of points), we calculate the observed event proportion in the training set, together with a 95% confidence interval using the exact binomial test (Supplementary Table S2). To assess model calibration, these proportions are compared to the observed event rates in the test set (Supplementary Figure S1).We used complete case analysis as missing data for the variables used were known to be rare and were assumed to be missing completely at random.

The study was reported in accordance with the TRIPOD and STROBE reporting guidelines [2, 20].

## Results

During the study period, 7,246 eligible patients with mild TBI were identified from the SUMC electronic health registry. Of those, 92 patients experienced CSDH during the follow-up period, as presented in Fig. 1. The statistical model is illustrated in Fig. 2.

Demographic and clinical features are presented in Table 1. The median age of participants at the time of head injury was 79 (IQR: 72–84). Forty percent were male, 34% had ischemic heart disease, 10% had renal failure, 30% were using antiplatelet medications, and 13% were using anticoagulants. Among the study participants, 9.5% had pathological head CT findings at the index event.

**Table 1:** Demographic and clinical characteristics of the study population

	Overall, N = 7,246^1^	CSDH, N = 92^1^	No CSDH, N = 7,154^1^	*p*-value^2^
Age	79 (72, 84)	81 (75, 85)	79 (72, 84)	0.01
Sex—Male	2,890 (40%)	60 (65%)	2,830 (40%)	< 0.001
Diabetes	2,935 (41%)	46 (50%)	2,889 (40%)	0.06
Ischemic heart disease	2,473 (34%)	39 (42%)	2,434 (34%)	0.09
Hypertension	5,108 (70%)	72 (78%)	5,036 (70%)	0.10
Renal Failure	724 (10.0%)	20 (22%)	704 (9.8%)	< 0.001
Dyslipidemia	4,668 (64%)	57 (62%)	4,611 (64%)	0.62
Heart Failure	994 (14%)	16 (17%)	978 (14%)	0.30
Atrial Fibrillation	1,270 (18%)	17 (18%)	1,253 (18%)	0.81
Antiplatelets	2,207 (30%)	34 (37%)	2,173 (30%)	0.17
Aspirin	2,045 (28%)	32 (35%)	2,013 (28%)	0.16
Clopidogrel	234 (3.2%)	5 (5.4%)	229 (3.2%)	0.22
Clopidogrel + Aspirin	88 (1.2%)	3 (3.3%)	85 (1.2%)	0.10
Anticoagulants	953 (13%)	18 (20%)	935 (13%)	0.07
Vitamin K antagonists	451 (6.2%)	5 (5.4%)	446 (6.2%)	0.75
Enoxaparin	38 (0.5%)	5 (5.4%)	33 (0.5%)	< 0.001
Direct oral anticoagulants	570 (7.9%)	10 (11%)	560 (7.8%)	0.28
Dabigatran	133 (1.8%)	3 (3.3%)	130 (1.8%)	0.24
Apixaban	347 (4.8%)	6 (6.5%)	341 (4.8%)	0.45
Rivaroxaban	118 (1.6%)	1 (1.1%)	117 (1.6%)	1.00
Pathological CT	686 (9.5%)	53 (58%)	633 (8.8%)	< 0.001

^1^Median (IQR); n (%)

^2^Wilcoxon rank sum test; Pearson’s Chi-squared test; Fisher’s exact test

*CSDH* Chronic subdural hematoma, *CT* Computed tomography

**Fig. 3:**
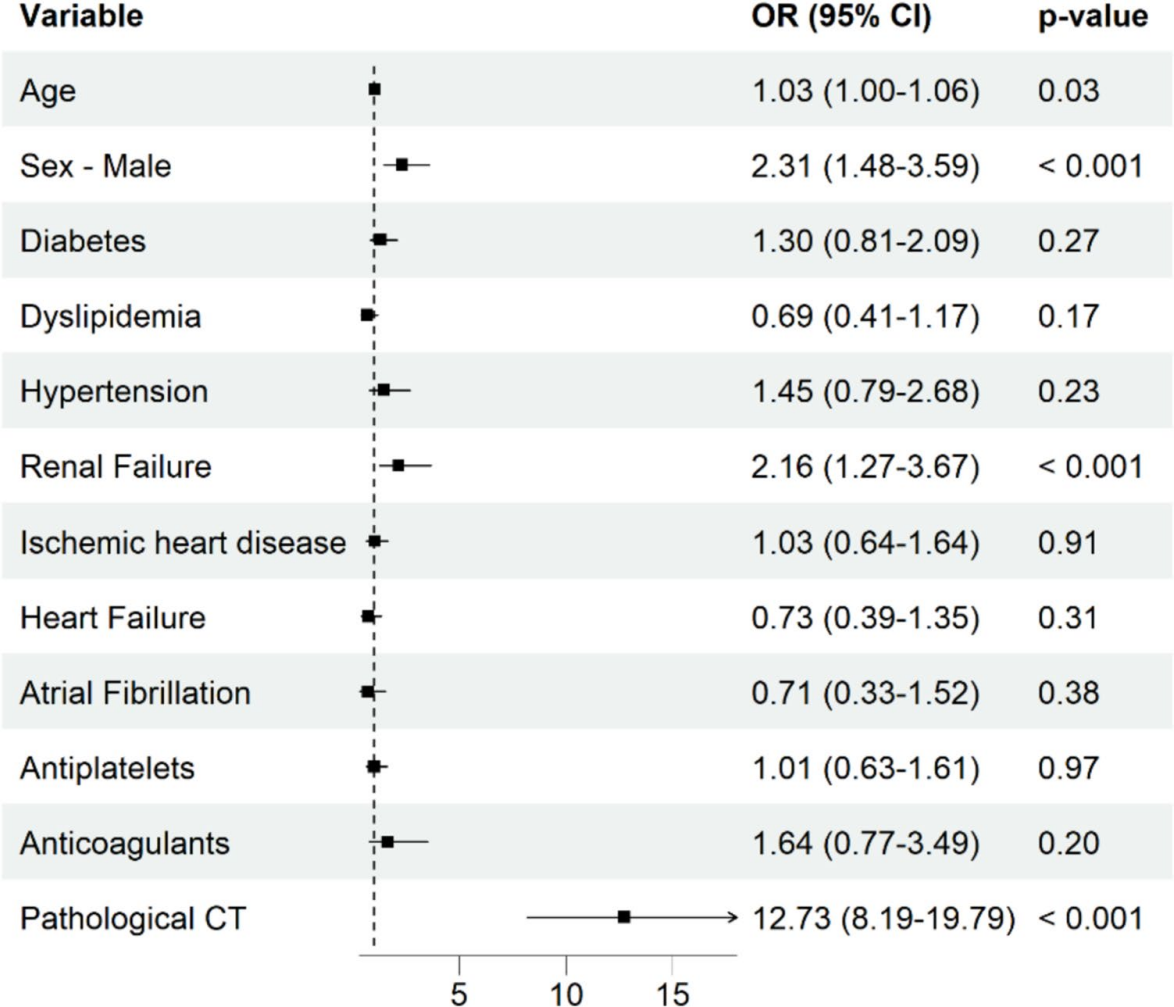
Adjusted odds ratios from a multivariable logistic regression model using GEE. Age was coded in 5-year intervals above 65 years. GEE: Generalized estimating equations; CSDH: Chronic subdural hematoma; OR: Odds ratio; CI: Confidence interval; CT: Computed tomography

A summary of the multivariable logistic regression model fit using GEE is presented in Fig. 3.

Age (per one-year) at TBI showed an OR of 1.03, 95% CI of 1.00–1.06. Male sex demonstrated an OR of 2.31, (95% CI: 1.48–3.59). Renal failure was associated with CSDH (OR 2.16, 95% CI: 1.27–3.67). Neither antiplatelet (OR 1.01, 95% CI: 0.63–1.61) nor anticoagulant (OR 1.64, 95% CI: 0.77–3.49) use showed a significant association with CSDH. Pathological CT findings at the index event demonstrated the strongest association (OR 12.73, 95% CI: 8.19–19.79). Other comorbidities such as diabetes, hypertension, ischemic heart disease, heart failure, and atrial fibrillation were not significantly associated with the development of CSDH.

LASSO regression selected the following features for the prediction model: pathological CT, male sex, renal failure, and age. Based on domain expertise, anticoagulant use was also included. Rounded estimates based on a GEE logistic regression model are presented in Table 2. A threshold of 5 points or higher was established to identify high-risk patients, aiming to achieve a sensitivity of 80%.

**Table 2:** A score-based risk model for CSDH following mild TBI, patients are considered high-risk if they score 5 points or higher

Predictor	Score
Age^1^	1
Sex—Male	4
Renal Failure	4
Anticoagulants	4
Pathological CT	14

^1^Number of points for every five years over 65 years

*CSDH* Chronic subdural hematoma, *TBI* Traumatic brain injury, *CT* Computed tomography

**Fig. 4:**
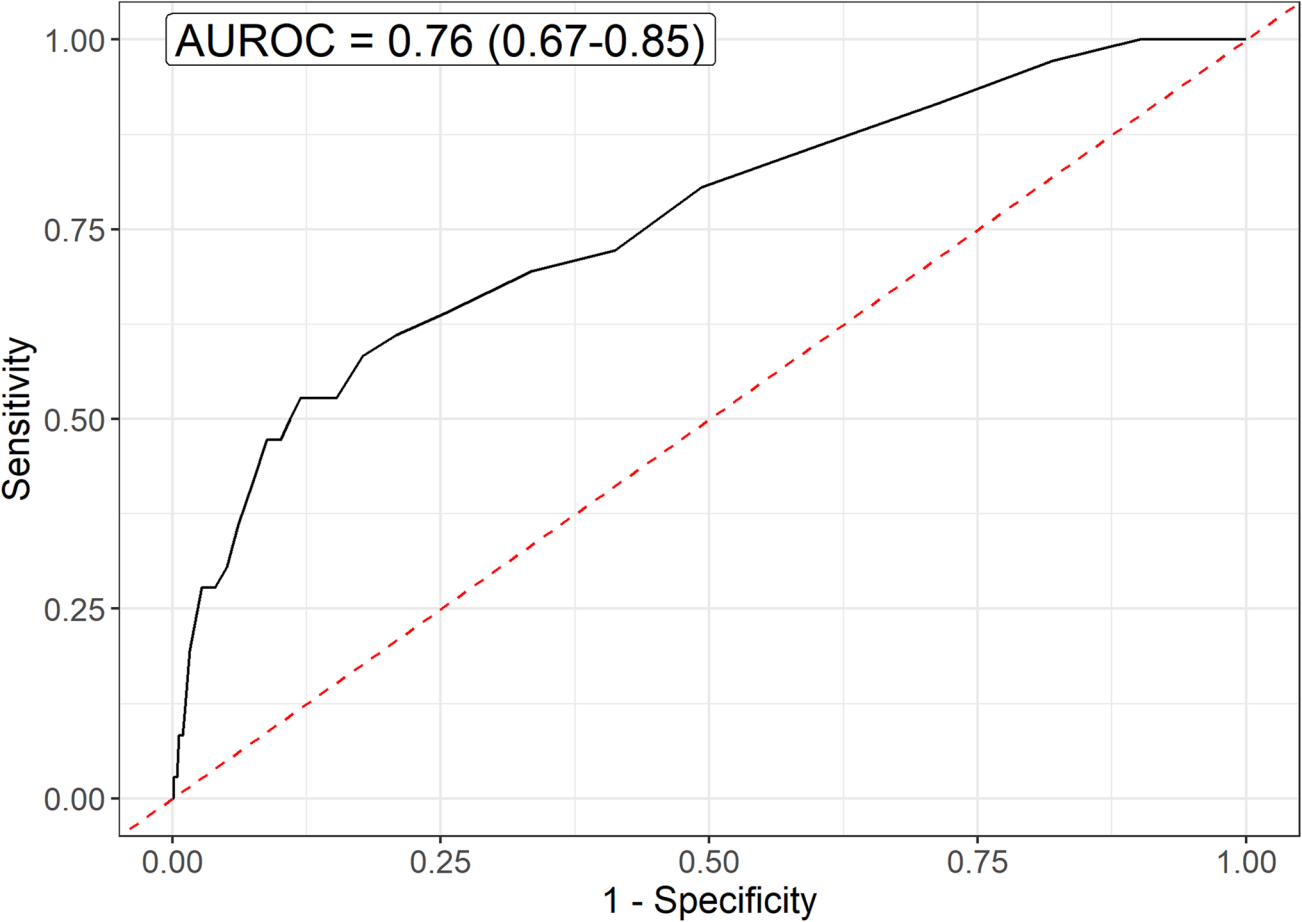
Receiver operating characteristic curve illustrating the performance of the score-based risk model for predicting chronic subdural hematoma in mild TBI patients on the independent test set. The model achieved an AUROC of 0.76 (95% CI: 0.67–0.85), indicating good discrimination between patients who do and do not develop CSDH. AUROC: Area under the receiver operating characteristics curve

**Fig. 5:**
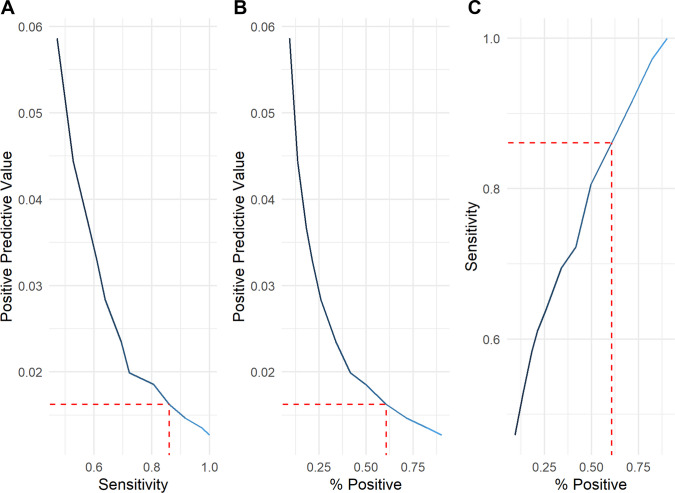
The three-panel plot depicts: (**A**) sensitivity vs. positive predictive value (PPV); (**B**) percentage of patients classified as high-risk vs. PPV; and (**C**) sensitivity vs. percentage of patients classified as high-risk. The red dashed lines indicate the predefined high-risk cutoff (score ≥ 5, Table 2), highlighting the corresponding sensitivity (~ 86%), PPV (~ 1.6%), and proportion of patients classified as high-risk (~ 60%)

The score-based risk model’s performance on the test set is presented in Figs. 4 and 5. The area under the receiver operating characteristic curve (AUROC) was 0.76 (95% CI: 0.67–0.85). Using the established high-risk threshold on the independent test set, the model achieved 86.1% sensitivity (95% CI: 70.5–95.3%) and a PPV of 1.6% (95% CI: 1.1–2.3%). The model classified 60.6% (95% CI: 58.9–62.3%) of patients as high-risk.

Observed event rates increased with higher total points scores, demonstrating overall good internal calibration of the risk score (Supplementary Table S2). The model demonstrated strong calibration, with observed probability of events remaining within the predicted confidence intervals per total points category (Supplementary Figure S1).

## Discussion

In this study, we first analyzed risk factors for CSDH following mild TBI in the elderly and then developed and validated a score-based risk model for bedside risk stratification. We found several risk factors associated with mild TBI, with the strongest being pathological CT findings at presentation. Our risk model was highly discriminatory and could potentially enable efficient bedside risk stratification, opening the possibility for targeted surveillance protocols in high-risk populations.

In our cohort, 9.5% of mild TBI patients had pathological CT findings, which was the strongest predictor for CSDH within 2–12 weeks post-injury. This observation contributes to the growing body of evidence suggesting that pathological CT findings during mild TBI events, first subgrouped as “complicated mild TBI” by Williams et al., may indicate a distinct clinical entity compared to “uncomplicated mild TBI” (CT-negative cases) [4, 21].

In this cohort, neither antiplatelet nor anticoagulant use was significantly associated with CSDH development after mild TBI. These findings differ from a previous case–control study by Gaist et al. on subdural hematoma cases, which reported that antithrombotic agents (both antiplatelet and anticoagulant agents) were associated with an increased risk of subdural hematomas, particularly the use of vitamin K antagonists combined with an antiplatelet drug. This discrepancy may be attributed to differences in study design and data sources [5]. Conversely, a Swedish study had found higher representation of patients using antithrombotic agents in non-traumatic CSDH, and no association between use of antithrombotic agents and recurrence of CSDH [9]. Similarly, Zhang et al., in a study comprising over 500 CSDH events, demonstrated a higher prevalence of non-traumatic CSDH in patients under antithrombotic medication [24]. These reports may explain the absence of an association between antithrombotic agents and CSDH development following traumatic brain injury observed in this study.

The clinical implications of antithrombotic agents remain an area of active investigation. For instance, Younsi et al., evaluating the need for reoperation in CSDH patients, demonstrated comparable risk across groups receiving anticoagulants, antiplatelet, or no antithrombotic medications [23]. While male sex remains a risk factor for CSDH without a definitive explanation, an Australian study comparing female versus male CSDH patients identified a stronger association between anticoagulant and antiplatelet therapy and female sex [11]. Further research is necessary to explore these distinctions.

According to the American College of Radiology, routine follow-up CT after an abnormal initial CT is supported for moderate to severe TBI and for patients on antiplatelets or anticoagulants [17]. However, the findings of this study support recognizing all patients with initial pathological CT findings as high-risk. For patients without an initial pathological CT, this study highlights the relevance of other risk factors, including older age, male sex, and renal failure. Although the association of anticoagulant use with CSDH was not statistically significant in the analysis, it was included in the score-based risk model due to established literature in the field [7, 14, 22].

The score-based model developed in this study achieved high sensitivity, identifying over 85% of the cases. Successfully identifying high-risk patients may provide a window of opportunity to perform a timely CT scan that could detect the hematoma in its subclinical phase. At this stage, conservative management strategies, including corticosteroid therapy or middle meningeal artery embolization (MMAE), may be considered as alternatives to surgical evacuation [14]. Furthermore, in cases where medical management fails, these patients could undergo controlled (non-emergent) neurosurgical intervention with proper preparation for both the procedure and anesthesia.

Although several individual predictors identified in this study have been previously described, our work examines these factors from the time of mild TBI onward, thereby addressing an important clinical decision point focused on post-discharge surveillance, rather than CSDH treatment. By focusing on the transition from mild TBI to delayed CSDH, this approach complements symptom-driven management of established CSDH by informing earlier risk stratification and follow-up planning in patients who are often discharged without scheduled surveillance. Furthermore, earlier identification of CSDH may allow for more deliberate clinical decision-making, including the possibility of planned rather than emergency intervention, as well as consideration of non-surgical treatment options such as MMAE. As described by Michael et al., MMAE has been proposed as a potentially less invasive treatment option in selected patients, particularly older individuals [12]. These considerations suggest a possible role for earlier surveillance and diagnosis, while acknowledging that further studies are needed to define clinical impact.

This study has several limitations. First, it is based on secondary data collected for clinical care. Such data could introduce coding errors, though clinical quality assurance was performed on a random subset of records by a specialist neurosurgeon. Second, although recent consensus criteria from the American Congress of Rehabilitation Medicine define mild TBI broadly (typically GCS 13–15) [18], our study employed a rigorous definition of mild TBI (GCS 14–15), excluding GCS 13 patients to specifically focus on the mildest end of the TBI spectrum, reflecting our institutional protocol where GCS ≤ 13 necessitates mandatory admission due to higher clinical suspicion. Third, this study does not directly consider neurosurgical interventions in CSDH patients. Finally, we may have been underpowered to detect more rare predictors of CSDH due to a limited sample size. The main strength of this study is the integration of clinical data from a large tertiary district health center with background and follow-up data from a nationwide insurer-provider in a country with mandatory health insurance.

In conclusion, we identified important risk factors for CSDH following mild TBI in elderly patients, including age, male sex, renal failure, and a pathological CT at initial presentation. Additionally, we present a concise, score-based prediction model with potential applications in ED risk stratification and follow-up of high-risk populations, though its clinical utility requires further investigation before implementation.

## Supplementary Information

Below is the link to the electronic supplementary material.

Supplementary file1 (DOCX 138 KB)

## Data Availability

No datasets were generated or analysed during the current study.
