# Supplementary material for: Predicting chronic subdural hematoma risk in elderly patients with mild traumatic brain injury
Source: Acta Neurochir (Wien). 2026 Feb 18;168(1):40. doi: 10.1007/s00701-026-06788-5 (PMC12920413; doi:10.1007/s00701-026-06788-5)
Supplement: Supplementary file 1 — Supplementary file1 (DOCX 138 KB) [file 701_2026_6788_MOESM1_ESM.docx]

## Supplementary

**Supplementary Table S1 – ICD-9 codes**

| **Diagnosis** | **ICD-9 code** |
| --- | --- |
| Traumatic brain injury | 9590[1,9], 920[0-9], 850[0-9], 851[0-9], 854[0-9] |
| Pathological CT finding at head injury event | 430, 431, 432, 801, 852, 4321,` 800[3-6], 801[0-3], 8037, 8043, 804[4,7], 852[4,5], 80039, 80049, 80059, 8006[0-1], 80109, 80119, 8012[4,9], 80130, 80419, 80429, 80439, 8524[0,1], 8525[1-6,9], 8003[0-6], 8004[0-6], 8005[0-6], 8010[0-6], 8011[0-6], 8012[0-6], 8041[1-6], 8042[4-6], 8043[1-6], 852[0-9], 853[0-9] |
| Chronic subdural hematoma | 432.1, 852, 852.2x, 852.3x |
| Burr hole procedure / Craniotomy / Craniectomy | Z0124, Z01240, Z0125, Z01311 |

**Table S1** ICD: International classification of diseases; CT: Computed tomography

**Supplementary Table S2 – Calibration, observed event percentage per predicted risk level (by number of total points)**

| **Points** | **95% CI of observed event percentage^1^** |
| --- | --- |
| **0** | 0.00-1.87% |
| **1** | 0.00-0.91% |
| **2** | 0.00-0.73% |
| **3** | 0.00-0.65% |
| **4** | 0.00-0.55% |
| **5** | 0.04-1.08% |
| **6** | 0.05-1.34% |
| **7** | 0.13-1.83% |
| **8** | 0.26-2.38% |
| **9** | 0.01-2.05% |
| **10+** | 2.99-5.38% |
| ^1^CI: Confidence interval, Exact binomial test | |

**Table S2** Predicted probability ranges for chronic subdural hematoma by point-based risk level, derived from observed event rates

**Supplementary Figure S1 – Calibration plot**


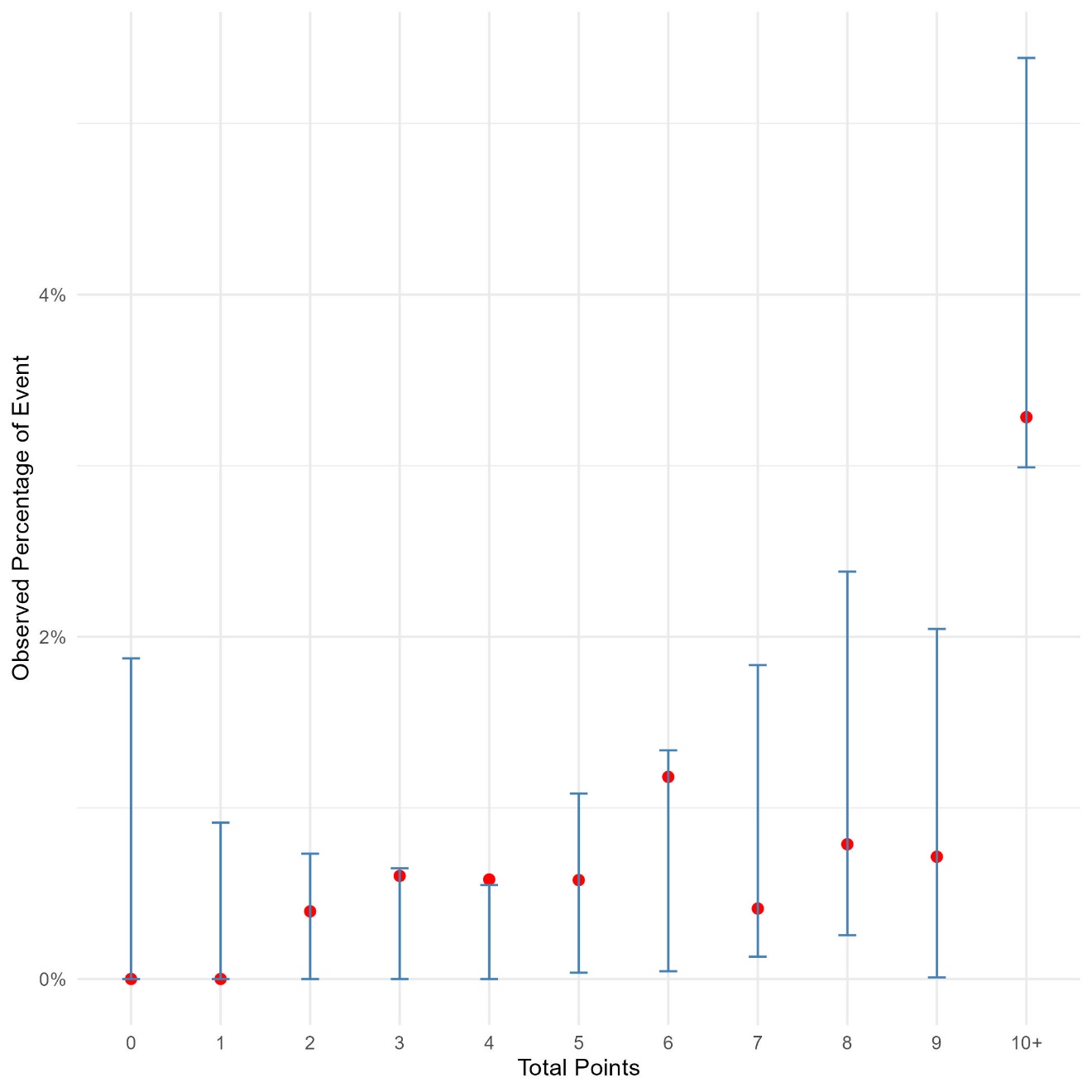


**Fig. S1** Calibration plot comparing predicted probability ranges of chronic subdural hematoma by point-based risk level (training set) with observed event rates in the test set
